# Supplementary material for: Incidence and Practice of Early Prone Positioning in Invasively Ventilated COVID-19 Patients—Insights from the PRoVENT-COVID Observational Study
Source: J Clin Med. 2021 Oct 19;10(20):4783. doi: 10.3390/jcm10204783 (PMC8541588; doi:10.3390/jcm10204783)
Supplement: Supplementary file 1 [file jcm-10-04783-s001.zip › jcm-1342301-supplementary.pdf]

Incidence and Practice of Early Prone Positioning in Invasively Ventilated COVID-19 Patients—Insights from the PRoVENT-COVID Observational Study

Willemke Stilma[1,2], David van Meenen[1], Christel M.A. Valk[1], Hendrik de Bruin[1], Frederique Paulus[1,2], Ary Serpa Neto[3,4] and Marcus J. Schultz[1,5,6] and on behalf of the PRoVENT-COVID † Collaborative Group ‡

1 Department of Intensive Care, Amsterdam UMC, location 'AMC', 1105 AZ Amsterdam, The Netherlands

2 Center of Expertise Urban Vitality, Faculty of Health, Amsterdam University of Applied Sciences, 1105 BD Amsterdam, The Netherlands

3 Australian and New Zealand Intensive Care Research Centre (ANZIC-RC), Monash University, 3004 Mel-bourne, Australia

4 Department of Critical Care Medicine, Hospital Israelita Albert Einstein, 05652-900 São Paulo, Brazil

5 Mahidol–Oxford Research Unit (MORU), Faculty of Tropical Medicine, Mahidol University, Bangkok 10400, Thailand

6 Nuffield Department of Medicine, University of Oxford, OX3 7BN Oxford, UK

Supplementary Materials of contents

Table S1. Univariable and Multivariable Model of Covariates Selected for Inclusion in the Final Model Mortality. 3

Table S2. Univariable and Multivariable Model linear mixed model initiation prone positioning. 4

Figure S1. Distribution curves 4 groups tidal volume, PEEP, driving pressure and compliance day 1. 5

Figure S2. Distribution curves 4 groups P/F ratio, FiO_2, PO_2, PCO_2 day 1. 6

Figure S3. Distribution curves 4 groups tidal volume, PEEP, driving pressure and compliance day 2. 7

Figure S4. Distribution curves 4 groups P/F ratio, FiO_2, PO_2, PCO_2 day 2. 8

Figure S5. Distribution curves 4 groups tidal volume, PEEP, driving pressure and compliance day 3. 9

Figure S6. Distribution curves 4 groups P/F ratio, FiO_2, PO_2, PCO_2 day 3. 10

Figure S7. Line graphs tidal volume, driving pressure, PEEP and compliance day 0, 1, 2, 3. 11

Figure S8. Line graphs P/F ratio, PO_2, PCO_2, FiO_2 for day 0, 1, 2, 3. 12

Figure S9. Outcomes. 13

Table S3. Time dependent cox regression analysis. 14

Table S1. Univariable and Multivariable Model of Covariates Selected for Inclusion in the Final Model Mortality.

	Univariable Model		Multivariable Model	
	**estimates**	***p* value**	**estimates**	***p* value**
**Covariates**				
PEEP	1.08 (0.94–1.24)	0.25		
Tidal volume per predicted bodyweight	0.99 (0.86–1.14)	0.90		
Severity ARDS	1.12 (0.98–1.29)	0.09	0.87 (0.74–1.03)	0.11
Body mass index	0.88 (0.76–1.05)	0.16	1.12 (0.97–1.29)	0.10
FiO_2	1.05 (0.92–1.20)	0.43		
NMBA	1.04 (0.79–1.35)	0.78		
'Early' prone positioning (day 0 or 1)	0.95 (0.83–1.09)	0.52		

Abbreviations: CI, confidence interval; FiO_2, fraction of inspired oxygen; NMBA, neuromuscular blocking agents; $PaCO_2$, arterial carbon dioxide tension; PaO_2, arterial oxygen tension.

Continuous variables were included after standardization and the hazard ratio represents the increase in one standard deviation of the variable.

Variables with a $P < 0.20$ were selected for inclusion in the multivariable model and variables with $P < 0.05$ in the multivariable model were selected for inclusion in the final model.

Table S2. Univariable and Multivariable Model linear mixed model initiation prone positioning.

	Univariable Model		Multivariable Model	
	**Estimates (95% CI)**	***p* value**	**Estimates (95% CI)**	***p* value**
**Covariates for prone positioning**				
PEEP	0.02 (-0.01–0.06)	0.16	0.01 (-0.03–0.05)	0.54
Severity ARDS	0.11 (0.08–0.14)	<0.001	0.08 (0.04–0.12)	**<0.001**
Body mass index	0.01 (-0.03–0.04)	0.66		
FiO_2	0.09 (0.06–0.12)	<0.001	0.07 (0.03–0.11)	**0.001**
$PaCO_2$	0.03 (-0.01–0.07)	0.11	0.15 (-0.02–0.05)	0.42

Abbreviations: CI, confidence interval; FiO_2, fraction of inspired oxygen; NMBA, neuromuscular blocking agents; $PaCO_2$, arterial carbon dioxide tension; PaO_2, arterial oxygen tension.

Continuous variables were included after standardization and the hazard ratio represents the increase in one standard deviation of the variable.

Variables with a $P < 0.20$ were selected for inclusion in the multivariable model.

Figure S1. Distribution curves 4 groups tidal volume, PEEP, driving pressure and compliance day 1.

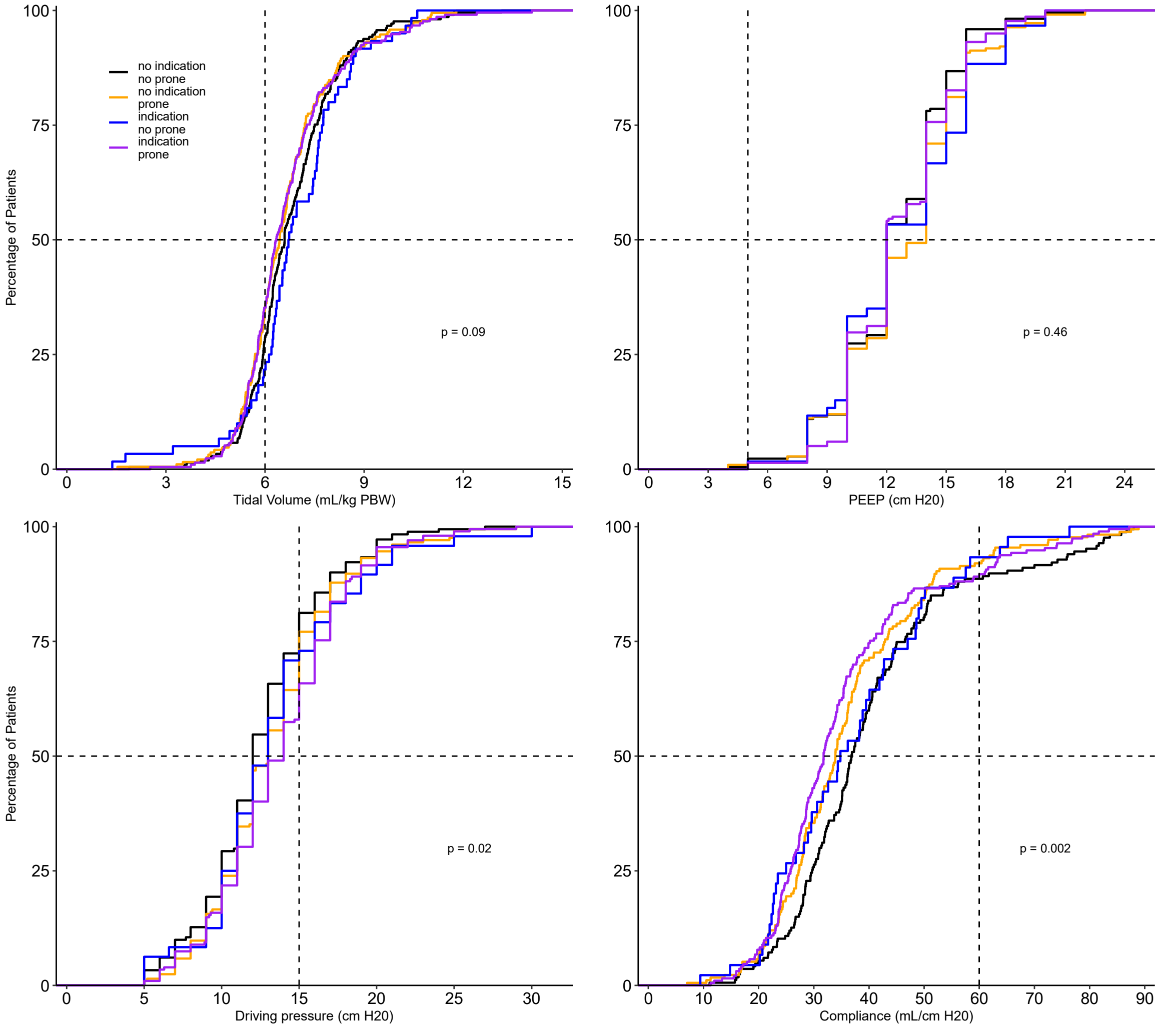

Figure S2. Distribution curves 4 groups P/F ratio, FiO2, PO2, PCO2 day 1.

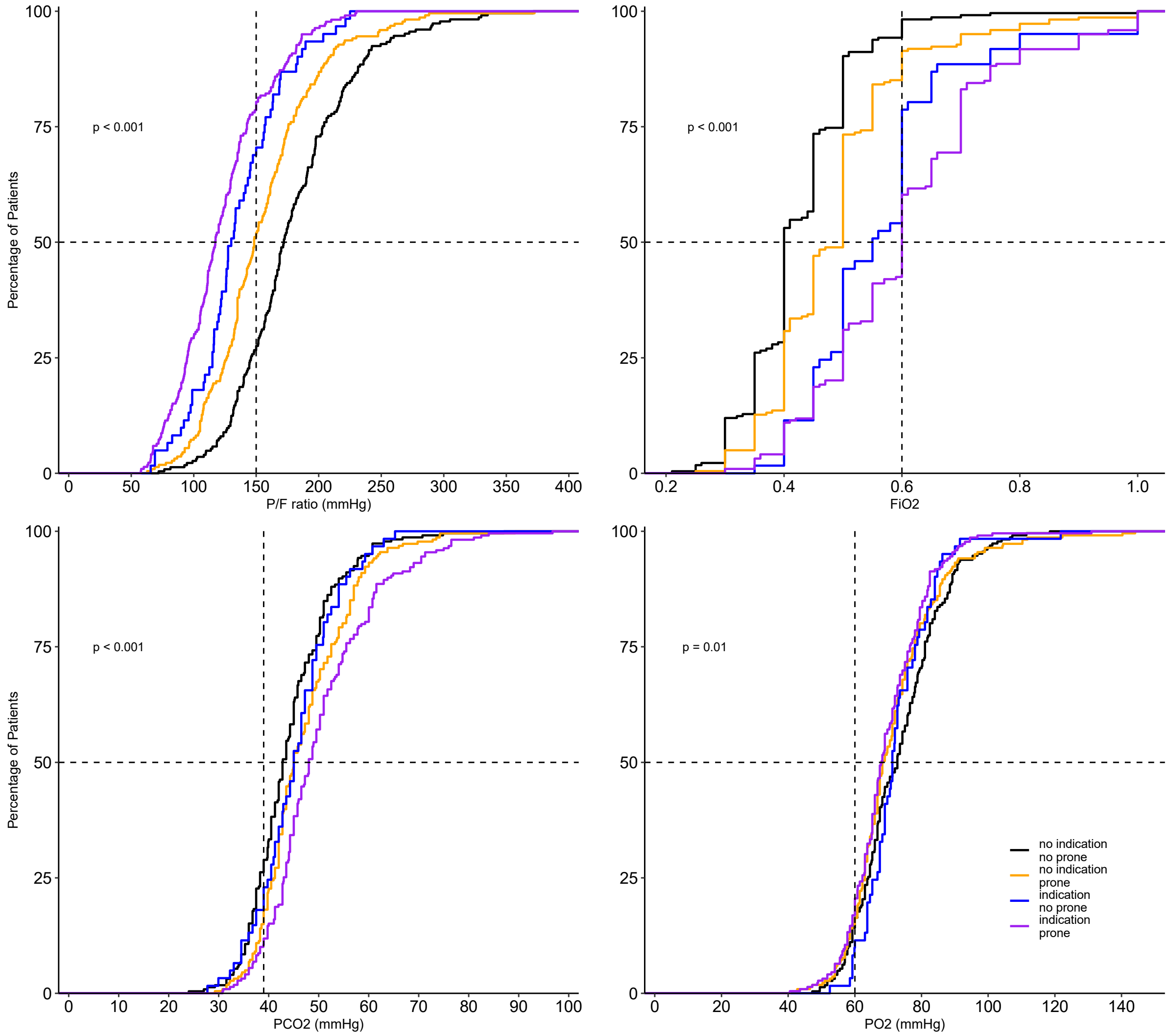

Figure S3. Distribution curves 4 groups tidal volume, PEEP, driving pressure and compliance day 2.

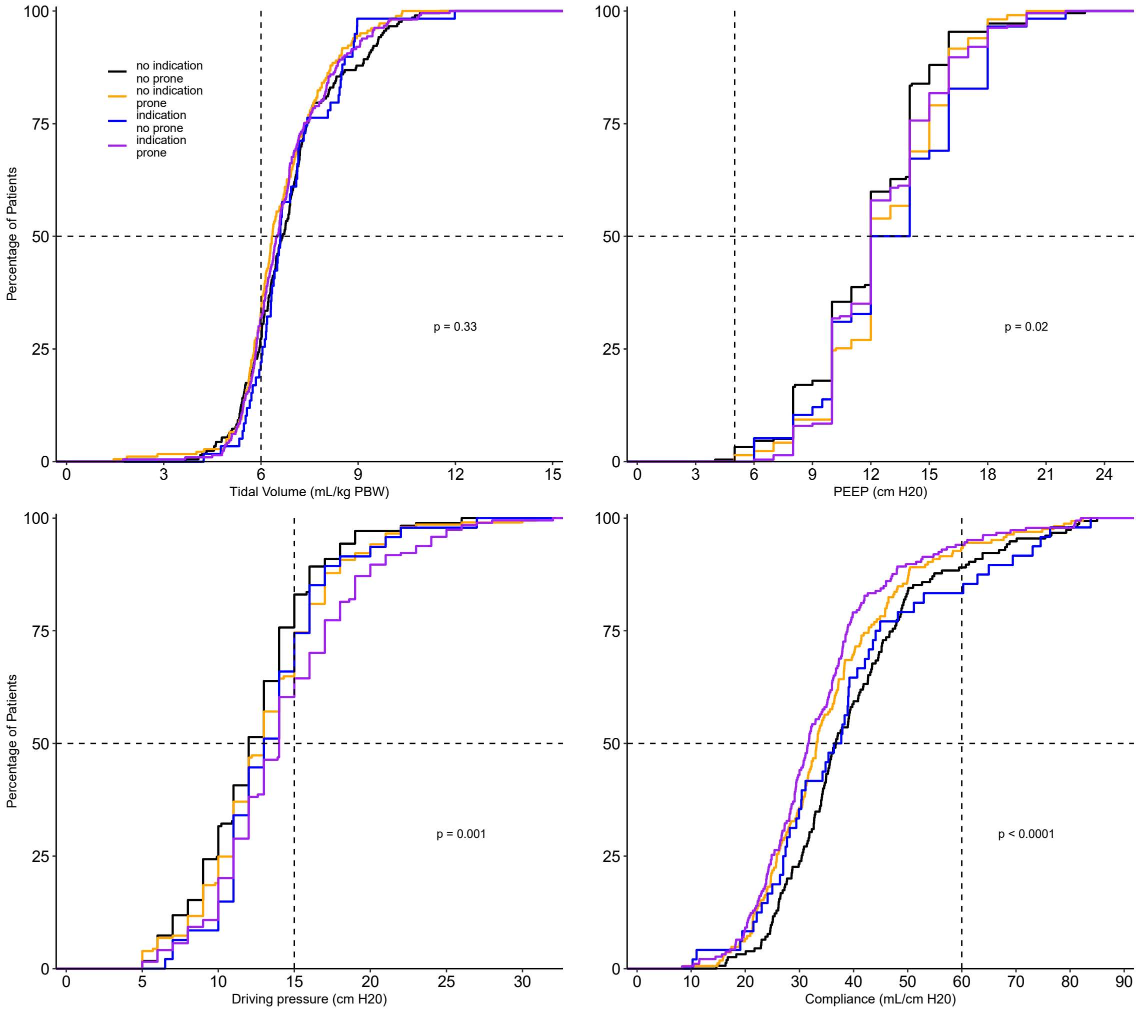

Figure S4. Distribution curves 4 groups P/F ratio, FiO$_2$, PO$_2$, PCO$_2$ day 2.

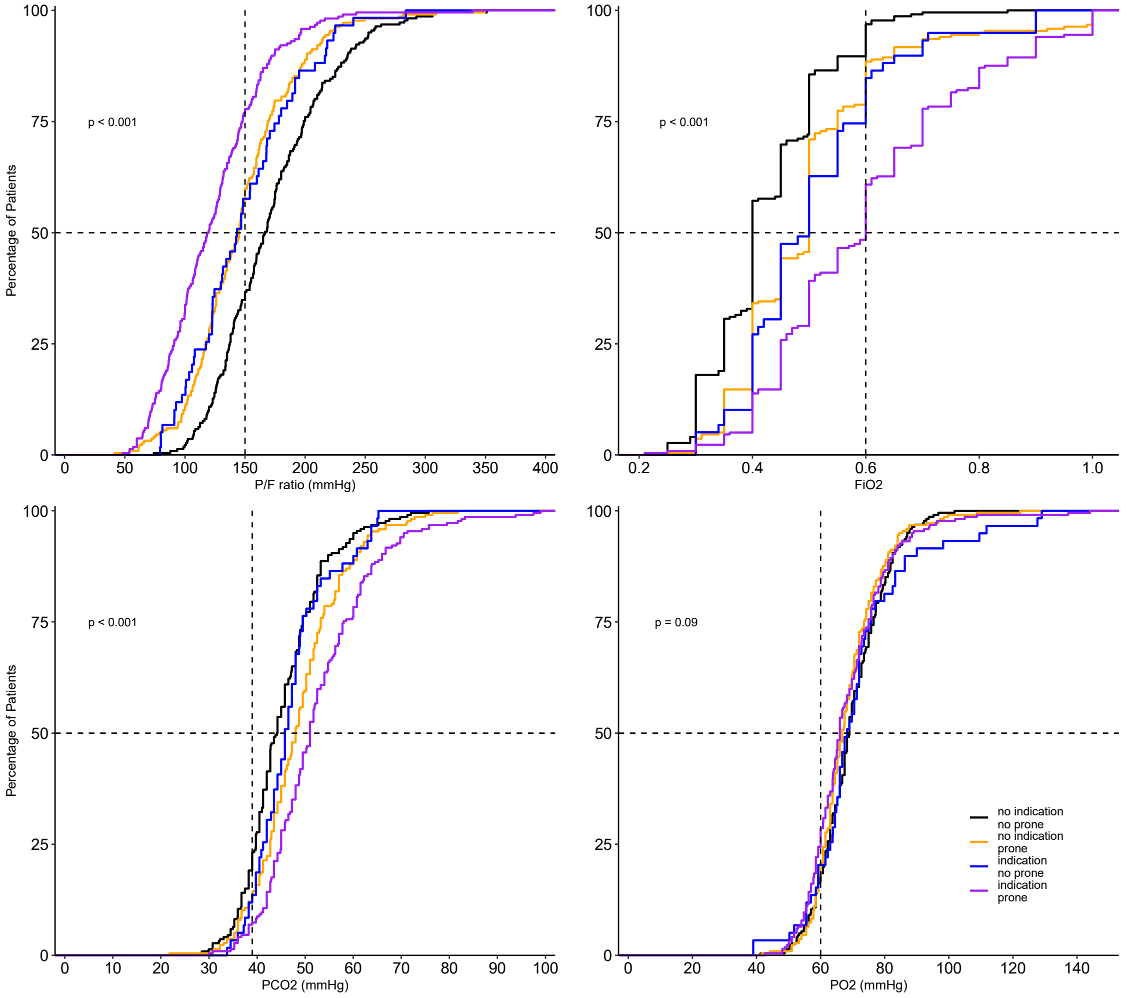

Figure S5. Distribution curves 4 groups tidal volume, PEEP, driving pressure and compliance day 3.

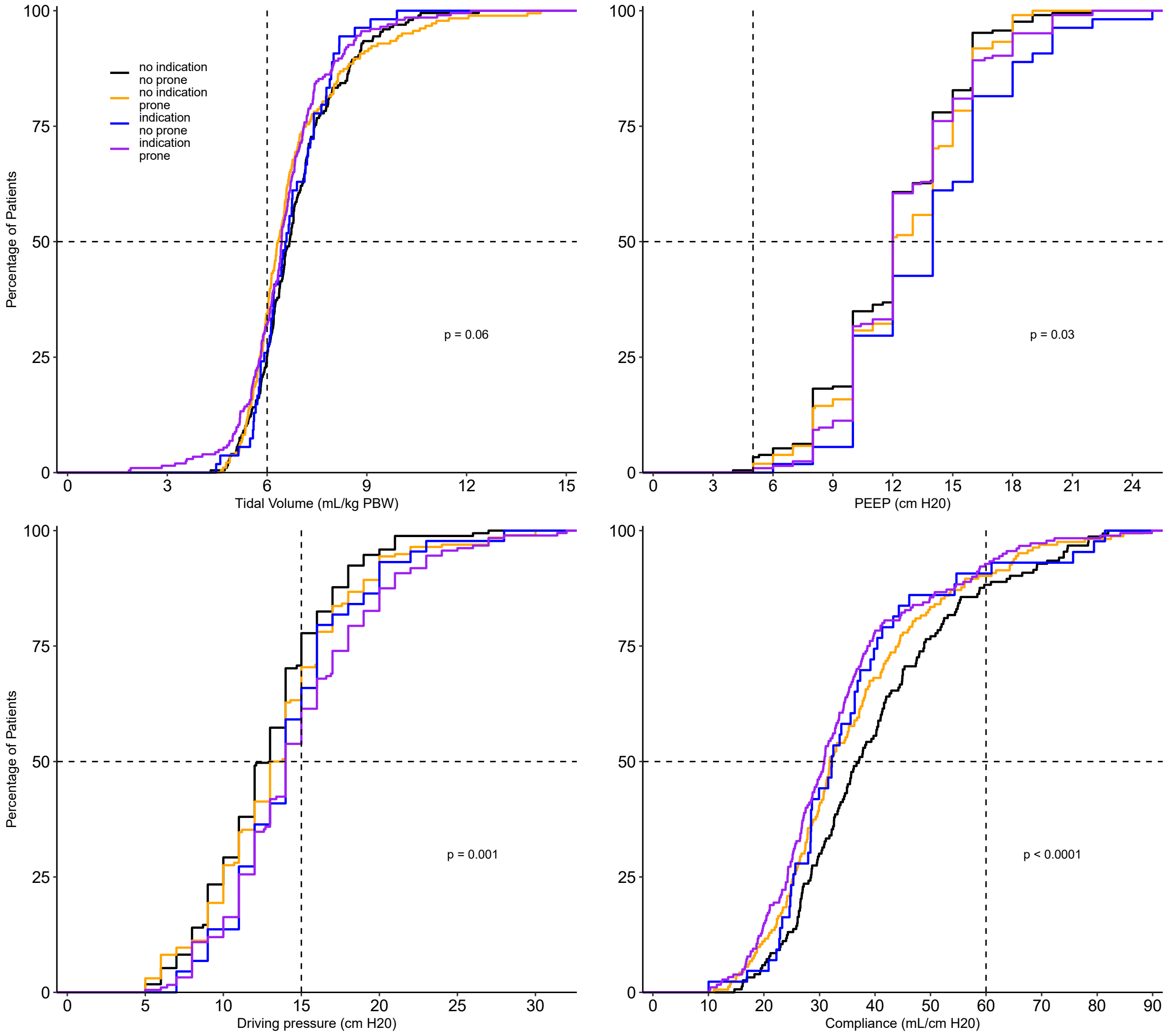

Figure S6. Distribution curves 4 groups P/F ratio, FiO_2, PO_2, PCO_2 day 3.

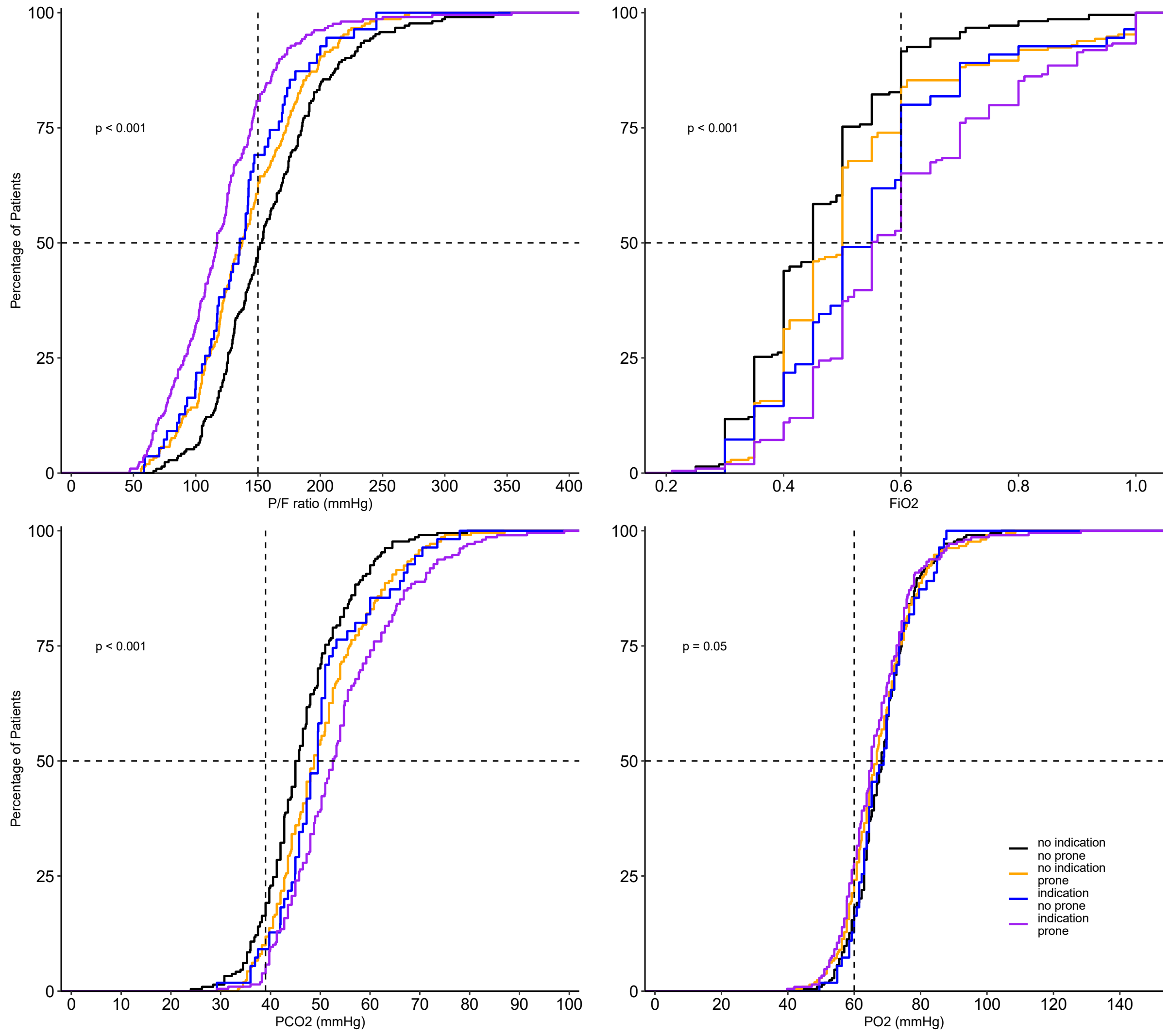

Figure S7. Line graphs tidal volume, driving pressure, PEEP and compliance day 0, 1, 2, 3.

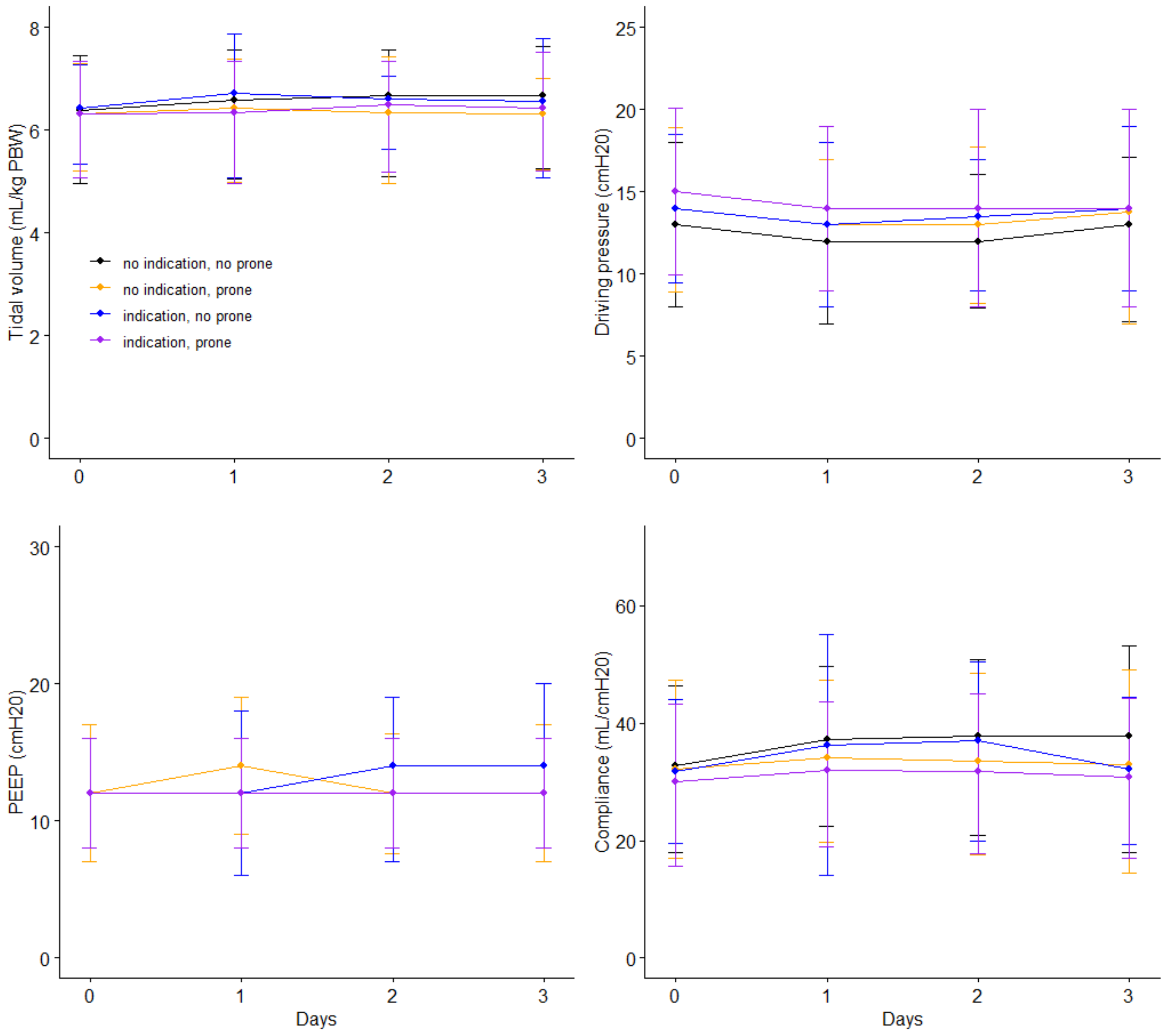

Figure S8. Line graphs P/F ratio, PO2, PCO2, FiO2 for day 0, 1, 2, 3.

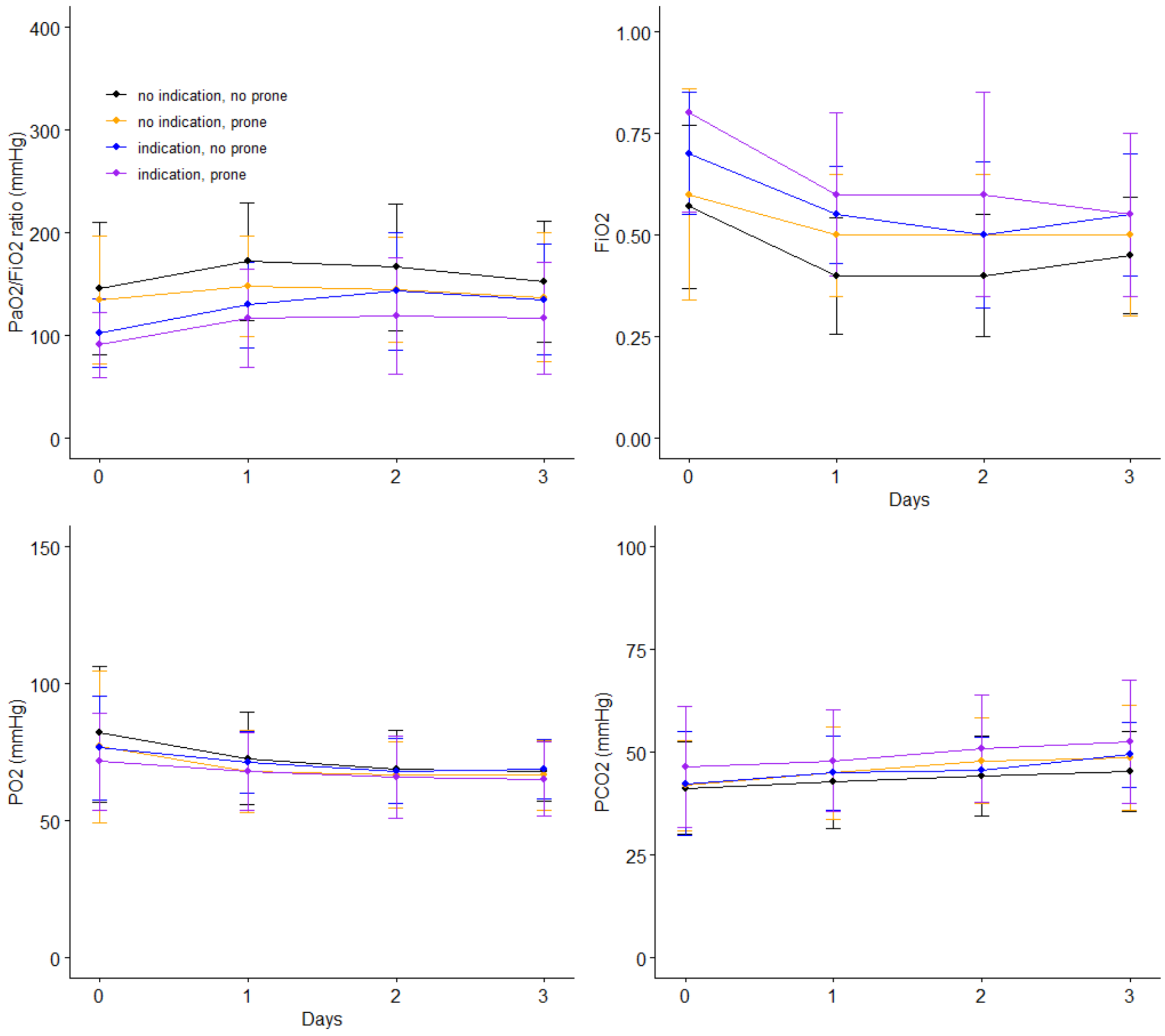

Figure S9. Outcomes.

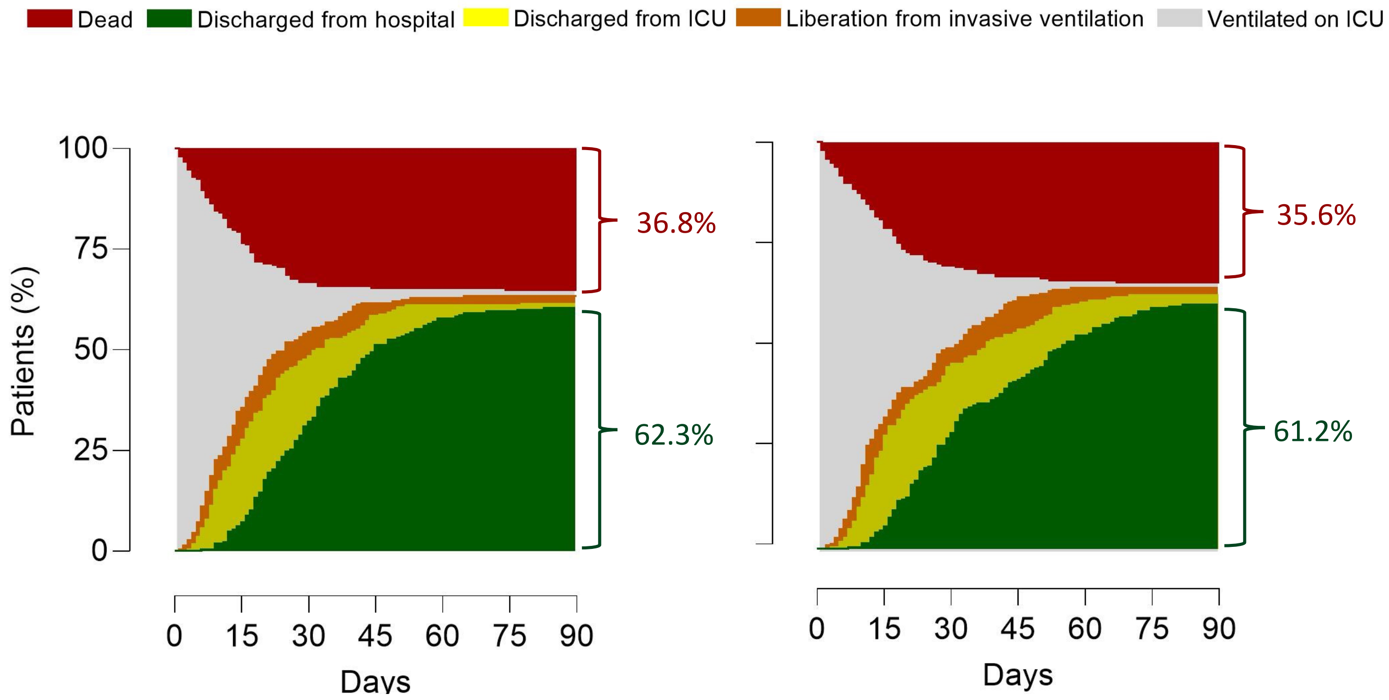

Patient outcomes for the groups of patients without an indication for prone positioning, on the left panel patients are displayed that did not receive prone positioning; on the right panel patients are displayed that did receive prone positioning.

HR's for outcomes were (no indication, no prone vs. no indication, prone vs. indication, no prone vs. indication, prone)

28-day mortality: 1.05 [0.76 – 1.45] vs. 0.88 [0.62 – 1.26] vs. 1.15 [0.80 – 1.54] vs. 0.96 [0.73 – 1.26] (P = 0.08)

90-day mortality: 0.93 [0.67 - 1.27] vs. 0.89 [0.64 - 1.24] vs. 1.19 [0.88 - 1.62] vs. 0.99 [0.76 - 1.28] (P = 0.02)

ICU discharge: 1.28 [1.02 – 1.61] vs. 1.03 [0.80 – 1.33] vs. 0.88 [0.69 – 1.12] vs. 0.89 [0.74 – 1.08] (P = 0.02)

Hospital discharge: 1.25 [0.99 – 1.58] vs. 1.07 [0.83 – 1.39] vs. 0.88 [0.69 – 1.13] vs. [0.89 [0.73 – 1.08] (P = 0.01)

Table S3. Time dependent cox regression analysis.

	Hazard Ratio's (95% CI)	*P* value
**Mortality day 28**		0.0027
Indication, no prone	1.46 (0.94–2.25)	
Indication, prone	1.20 (0.89–1.61)	
No indication, no prone	0.95 (0.70–1.27)	
No indication, prone	0.75 (0.55–1.02)	
**Mortality day 90**		0.0075
Indication, no prone	1.56 (1.05–2.34)	
Indication, prone	1.22 (0.93–1.61)	
No indication, no prone	0.88 (0.67–1.72)	
No indication, prone	0.77 (0.58–1.02)	
